# Bacterial contamination of platelets for transfusion: strategies for prevention

**DOI:** 10.1186/s13054-018-2212-9

**Published:** 2018-10-27

**Authors:** Jerrold H. Levy, Matthew D. Neal, Jay H. Herman

**Affiliations:** 1 Duke University Hospital, 2301 Erwin Road, Durham, NC 27710 USA; 2 University of Pittsburgh Medical Center, 200 Lothrop Street, Pittsburgh, PA 15213 USA; 3 Thomas Jefferson University Hospital, 111 S. 11th Street, Philadelphia, PA 19107 USA

**Keywords:** Bacterial contamination, Bacterial detection, Hemovigilance, Pathogen reduction/inactivation, Platelets, Prevention strategies, Septic transfusion reaction (STR), Transfusion, Transfusion-transmitted bacterial infection (TTBI)

## Abstract

Platelet transfusions carry greater risks of infection, sepsis, and death than any other blood product, owing primarily to bacterial contamination. Many patients may be at particular risk, including critically ill patients in the intensive care unit. This narrative review provides an overview of the problem and an update on strategies for the prevention, detection, and reduction/inactivation of bacterial contaminants in platelets. Bacterial contamination and septic transfusion reactions are major sources of morbidity and mortality. Between 1:1000 and 1:2500 platelet units are bacterially contaminated. The skin bacterial microflora is a primary source of contamination, and enteric contaminants are rare but may be clinically devastating, while platelet storage conditions can support bacterial growth. Donor selection, blood diversion, and hemovigilance are effective but have limitations. Biofilm-producing species can adhere to biological and non-biological surfaces and evade detection. Primary bacterial culture testing of apheresis platelets is in routine use in the US. Pathogen reduction/inactivation technologies compatible with platelets use ultraviolet light-based mechanisms to target nucleic acids of contaminating bacteria and other pathogens. These methods have demonstrated safety and efficacy and represent a proactive approach for inactivating contaminants before transfusion to prevent transfusion-transmitted infections. One system, which combines ultraviolet A and amotosalen for broad-spectrum pathogen inactivation, is approved in both the US and Europe. Current US Food and Drug Administration recommendations advocate enhanced bacterial testing or pathogen reduction/inactivation strategies (or both) to further improve platelet safety. Risks of bacterial contamination of platelets and transfusion-transmitted infections have been significantly mitigated, but not eliminated, by improvements in prevention and detection strategies. Regulatory-approved technologies for pathogen reduction/inactivation have further enhanced the safety of platelet transfusions. Ongoing development of these technologies holds great promise.

## Background

The safety of allogeneic blood products has increased over time because of significant improvements in donor screening, testing, and deferrals [1]. Nonetheless, potential risks of infection from viral, bacterial, and other pathogens remain [1, 2]. Bacterial contamination of platelets is a leading infectious risk of transfusion (Table 1 [3, 4]), and platelet transfusions are associated with a greater risk of sepsis and death than any other blood product [4]. The US Food and Drug Administration (FDA) has issued draft guidance recommending additional strategies to mitigate bacterial contamination of platelet transfusions [4].

**Table 1:** Bacterial species identified in platelet concentrates and implicated in transfusion-transmitted bacterial infections

Gram-positive	Gram-negative
Bacillus species^a^	*Klebsiella species*
	*Serratia species* ^a^
Streptococcus species	*Escherichia coli* ^a^
Staphylococcus species^a^	*Acinetobacter species*
	*Enterobacter species*
Propionibacterium acnes	*Providencia rettgeri*
	*Yersinia enterocolitica*

^a^Some of which are biofilm-producing species

Platelet transfusions are common in critically ill intensive care unit (ICU) patients [5], occurring in 9–30% of patients [6]. Platelets are also commonly transfused in a perioperative setting as either prophylaxis before surgery or treatment for bleeding during or after surgery.

This narrative review provides an update on strategies. We initially searched PubMed in December 2017 using the primary search phrase “(bacteria OR bacterial) AND (contamination OR contaminant) AND (platelet OR platelets)”. This initial search returned 510 results. We also performed searches with additional key terms (for example, “prevention”, “detection”, “biofilm”, “hemovigilance”, “surveillance”, “pathogen reduction”, and “pathogen inactivation”) to identify articles specifically relevant to each section of this review. Only English language, peer-reviewed articles were considered; no constraints on publication type or date were imposed. Titles/abstracts of retrieved articles were checked for relevance, and other relevant papers were identified by manual searching of reference lists and the authors’ personal literature collections. Where multiple articles reported similar findings, priority was given to those most recently published. In total, 70 articles were deemed by the authors as most relevant to the topic of bacterially contaminated platelets intended for transfusion and were included in this narrative review.

## Bacterial contamination of platelets

Skin microflora is a principal source of bacterial contamination. Furthermore, storage conditions—for example, gas-permeable bags at room temperature (20–24 °C) with continuous agitation—can effectively support bacterial growth. A significant proportion of bacterial species that contaminate platelets can form biofilms, multicellular aggregations often encased in an extracellular matrix that can adhere to biological and non-biological surfaces and evade detection by culture screening systems that are based on sampling of the supernatant [7]. Additionally, a 5-year study of over 2 million US platelet donations suggested that the risk of bacterial contamination and sepsis may be influenced by the type of plateletpheresis collection technology used [8, 9].

Transfusion-transmitted bacterial infection (TTBI) and septic transfusion reactions (STRs) are major sources of morbidity and mortality following platelet transfusion. In the US, the therapeutic adult dose of platelets is a single unit (that is, bag) which generally contains at least 3.0 × 10^11^ platelets [10]. The reported frequency of bacterial contamination of platelets ranges from 1:1000 to 1:2500 units [2, 11–13]. Consideration of the per-patient—rather than per-unit—risk provides visibility into the potential impact on patient outcomes. A review of four large-scale independent studies showed that for a hematology/oncology (H/O) patient receiving a mean of 6 apheresis platelet units per treatment episode, 1 out of 250 was at risk of receiving a contaminated platelet and 1 out of 1000 of having an STR [2]. In surgical patients, per-patient risk may differ given the number of platelet units transfused. Ning et al. observed a median of 1 platelet transfusion (interquartile range of 1–2) per ICU patient admission [14]. Greinacher and Selleng [15] noted that transfusion of 1 platelet unit is generally not sufficient for the thrombocytopenic ICU patient.

It has been acknowledged that TTBIs and STRs historically have been underreported [4]. The criteria used to identify these events may impose limitations on recognition. According to AABB (formerly the American Association of Blood Banks) and Centers for Disease Control and Prevention (CDC) criteria, TTBI can be concluded where no other potential confounding conditions exist, infection manifests within 24 h of transfusion, and a positive culture result is obtained from both patient samples and transfusion bag. However, transfusion bags are typically discarded after use and not available for culture, and patients on antibiotic therapy may not manifest positive blood cultures, making the criteria ineffective. Furthermore, common transfusion reactions that are not usually considered serious, such as febrile non-hemolytic transfusion reactions, often are not documented. Only symptomatic TTBIs during transfusion are recorded [11], yet infections may have variable times to manifest (that is, post-transfusion), especially in seated prosthetic material. For example, in a multistate US outbreak of *Pseudomonas fluorescens* bacteremia traced back to contaminated heparinized saline intravenous flush syringes, 41% of patients were diagnosed 84–421 days after the last potential exposure to a contaminated saline flush syringe [16].

Several studies have shown that common hemovigilance (HV) strategies—intended to collect, assess, and address information on unexpected or undesirable effects of blood products [17]—rarely detect TTBI-related morbidity and mortality, particularly when passive surveillance is employed. Passive surveillance relies on accurate and timely reporting of suspected transfusion-associated adverse reactions (often by untrained personnel) and can lead to underreporting [11, 18]. Alternatively, active surveillance strategies, which are not the standard for US HV, use trained individuals to search for and identify adverse reactions using standard definitions (sometimes with independent adjudication), and sampling and testing of blood products are carried out at the time of issue [11, 18]. Active surveillance, however, also has limitations, such as the use of aerobic culture only and the absence of methods addressing biofilm-forming organisms.

STRs are also underreported, as they may easily be missed in neutropenic patients or those on antibiotic therapy, because of passive surveillance strategies and limitations of current detection methods. Narrow and variable STR definitions which include fever and possibly other signs and symptoms (for example, rigors, tachycardia, and dyspnea) also lead to underreporting [11], especially when clinical features mimic alternative diagnoses [19]. In a retrospective study of over 50,000 platelet transfusions at a single large academic medical center, 20 out of 51,440 (0.04%, or 389 per million) platelet units were identified by culture-based active surveillance as being bacterially contaminated and resulted in five STRs, one of which was fatal and none of which was reported by passive surveillance to the hospital blood bank.

TTBIs and STRs can be life-threatening. The CDC recently reported on three patient deaths due to transfusion of bacterially contaminated platelets in Utah and California [20], adding to others that have been reported to the FDA [21]. In addition to the direct link between contaminated platelets and TTBIs and STRs, data suggest associations between platelet transfusion and bacterial infection incidence [5, 9].

## Strategies to mitigate risk of bacterial contamination

Different strategies can be used to reduce the incidence of bacterial infections and sepsis associated with platelet transfusions. Transfusion medicine has traditionally relied on methods designed to help avoid bacterial contamination at the time of blood collection, processing, and transfusion. The AABB’s Standards for Blood Banks and Transfusion Services, the guidepost for blood collection, processing, and administration, require that AABB-accredited facilities “have methods to limit and to detect or inactivate bacteria in all platelet components” [22]. These strategies are discussed below.

### Prevention of bacterial contamination at time of platelet collection

Donor selection is a first-line preventive measure and relies on assessing possible bacterial infections by evaluating the donor’s current medical conditions and antibiotic treatment. The antecubital fossa of donors is inspected to avoid venipuncture through scar tissue that might increase contamination. Donors are asked about signs of infection or illness. However, a survey of more than 11,000 donors suggests that responses may vary depending on how questions are asked: affirmative responses regarding gastrointestinal symptoms (a risk factor for *Yersinia* species contamination) were given by 0.6% or 4.0% of donors, depending on which one of two questions were asked [23]. Questionnaires rely on accurate donor recollection and symptom reporting, which may not always be sufficiently reliable and cannot identify asymptomatic bacteremia.

Skin flora is a primary source of bacterial contamination, and needles used for venipuncture may generate a small skin plug. Diversion of the initial blood volume (for example, 10–20 mL) reduces bacterial contamination of collected blood [24]. This procedure is effective in preventing Gram-positive bacterial infections caused by the skin bacterial microflora at the time of venipuncture. Blood collection using a diversion pouch is a standard practice to reduce contamination risk further [24].

### Testing methods for platelet bacterial contamination

#### Culture-based

Until recently, using culture-based methods to detect bacteria within prespecified hours of platelet collection has been the predominant method used by US blood collection establishments to comply with the AABB Standards for Blood Banks and Transfusion Services [22]. However, the sensitivity of primary bacterial culture screening is only 22–40% [12, 13, 25], detecting between 1 and 10 colony-forming units per milliliter [26]. Furthermore, the early sampling required to allow for microbial growth in a culture-based system is at high risk of sampling error due to the small starting number of contaminating bacteria in a platelet donation, particularly for slow-growing species [26]. Conventionally, culture-based detection methods focus on aerobic species. As a result, anaerobic, facultative, and fastidious bacteria may go undetected, especially when present at low concentrations. BacT/ALERT^®^(BioMérieux, Marcy-l’Étoile, France), which is FDA-approved for platelet quality control [26], is the primary automated culture system used in the US today for platelet screening after collection. This system uses CO_2_ levels to indicate bacterial growth and has been validated for detection of bacterial contaminants [27, 28]. Screening programs for bacterial contamination of platelet units using BacT/ALERT^®^ have shown efficacy [29] and reduced the incidence of TTBI [30]. The sensitivity of primary testing with this system was improved when platelet sampling was performed using a proportional sample volume (at least 3.8% of the collection volume) rather than a fixed sample volume; this may reduce sampling error and the need for secondary testing [31]. A disadvantage of current automated culture systems is that they may be ineffective at detecting biofilm-producing organisms [32].

#### Secondary rapid detection methods

Other techniques target components of the bacterial cell wall/membrane or intracellular molecules to detect contaminating bacteria [26]. The BacTx^®^ assay (Immunetics, Marlborough, MA, USA) detects bacterial peptidoglycan through colorimetric measurements [33] and is FDA-cleared for detecting bacterial contamination in platelets. Analytical sensitivity of BacTx is 10^3^ to 10^4^ [26]. The Platelet Pan Genera Detection (PGD) Test (Verax Biomedical, Marlborough, MA, USA), an immunoassay that detects bacterial lipopolysaccharide and lipoteichoic acid, has also demonstrated effectiveness [34, 35]. It is the only test FDA-approved as a point-of-issue safety measure in addition to a quality control test. Analytical sensitivities of the Platelet PGD Test are 10^3^ to 10^4^ for Gram-positive bacteria and 10^3^ to 10^5^ (some >10^6^) for Gram-negative bacteria [26]. The Platelet PGD Test is indicated for use within 24 h of transfusion of the tested platelet unit. When used with an appropriate FDA-approved platelet collection, processing, and storage system, the Platelet PGD Test can be used to extend platelet shelf life to 7 days from 5 days by testing within 24 h of transfusion on day 6 or 7 (or both). Disadvantages of secondary rapid detection methods include challenges with discordant results (false-positive rate of 0.51%) [36] as well as time, labor, and inventory management requirements associated with the testing paradigm.

## Pathogen reduction/inactivation technologies

**Fig. 1:**
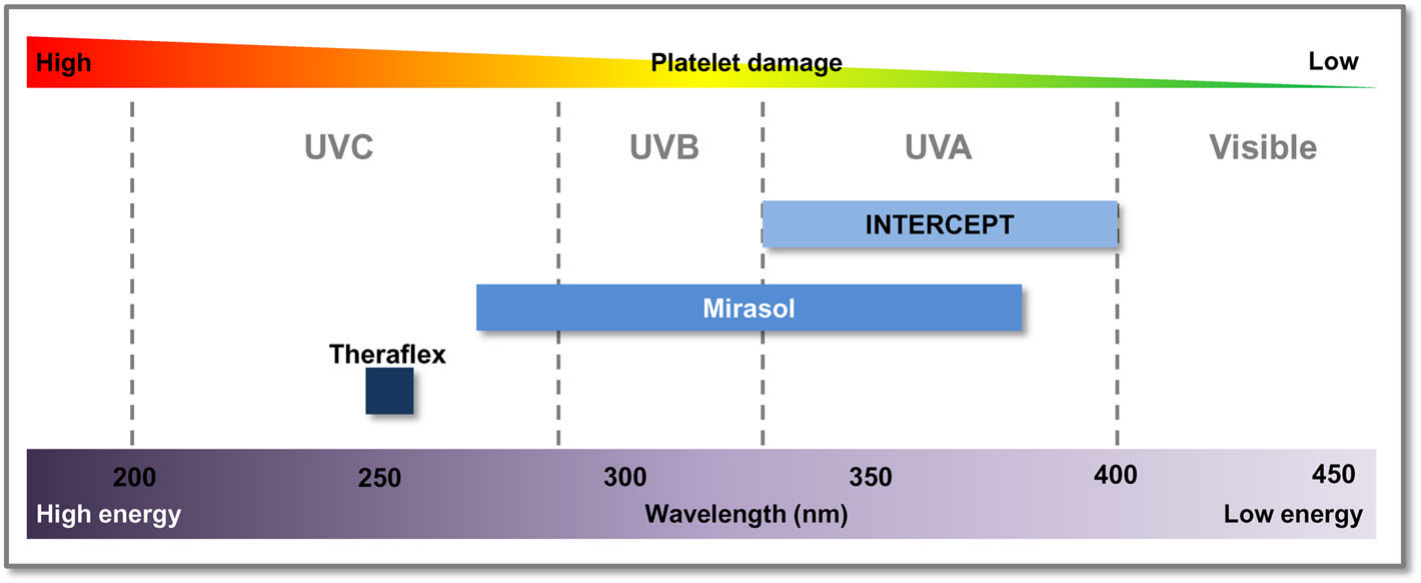
Wavelength, energy, and dose for pathogen reduction/inactivation technologies compatible with platelets. Irradiation doses for each technology are 3 J/cm^2^ (INTERCEPT), 6.2 J/mL (Mirasol), and 0.2–0.3 J/cm^2^ (THERAFLEX)

In contrast to reactive screening and detection strategies, newer technologies enable a proactive approach to reducing contaminants in blood components and preventing transfusion-transmitted infections. These technologies not only target bacteria but also mitigate risks associated with known and unknown viruses, parasites, protozoa, and leukocytes. Although some authors view the cost of these pathogen inactivation technologies as prohibitive [37, 38], they can be cost-effective and comparable with other blood safety interventions [39, 40] and are in universal application for all platelet components in France, Switzerland, and Belgium [41]. Recent developments for the reduction or inactivation (or both) of pathogens in blood products are based on using ultraviolet (UV) light. Three pathogen reduction/inactivation technologies are compatible with platelets (Table 2) [42–44]. Their mechanisms of action differ by the wavelength of UV light used, with concomitant differences in energy levels imparted: with shorter wavelengths, energy level increases, as does the potential to cause cellular damage (for example, to platelets) (Fig. 1) [43].

**Table 2:** Overview of pathogen reduction/inactivation technologies compatible with platelet concentrates

	INTERCEPT™ Blood System for Platelets	Mirasol^®^ Pathogen Reduction Technology System	THERAFLEX^®^ UV-Platelets
Manufacturer	Cerus Corporation	Terumo BCT	MacoPharma
FDA approval for platelets	Yes	No	No
CE mark approval	CE class III	CE class IIB	CE class IIB
Principle of method	UVA illumination in the presence of a photosensitizer	Broad-spectrum UV illumination in the presence of a photosensitizer	UVC illumination and intense platelet bag agitation
Photosensitizer	Amotosalen	Riboflavin	None
UV wavelength and dose	UVA, 320–400 nm, 3 J/cm^2^	UVB/UVA/UVC (100%/60%/20%), 265–370 nm, 6.2 J/mL	UVC, 254 nm, 0.2–0.3 J/cm^2^
Pathogens targeted	Bacteria (Gram-positive and Gram-negative), viruses (enveloped and non-enveloped), parasites	Bacteria (Gram-positive and Gram-negative), viruses (enveloped and non-enveloped), parasites	Bacteria (Gram-positive and Gram-negative), viruses (enveloped and non-enveloped), parasites
Toxicology testing^a^	Acute toxicology, carcinogenicity, general pharmacology, genotoxicity, phototoxicity, repeated dose, reproductive toxicology (plus others)	Acute toxicology, genotoxicity, phototoxicity, repeated dose, reproductive toxicology (plus others)	Not applicable (no exogenous photosensitizer)
Bacterial inactivation (log reduction)^b^			
Gram-positive	3.6 to >6.9	1.9 to 4.8	4.3 to >4.9
Gram-negative	4.5 to >6.7	2.8 to 5.4	>4.0 to >4.9
Maximum approved storage^c^	5 and 7 days	7 days	5 days

Abbreviations: *CE* Conformité Européene (“European Conformity”), *FDA* US Food and Drug Administration, *UV* ultraviolet

^a^See [43, 44] for additional details

^b^See [43, 44] for data on individual bacterial species tested

^c^Depending on country

### INTERCEPT™ Blood System for Platelets

The INTERCEPT™ Blood System for Platelets (Cerus Corporation, Concord, CA, USA) was first approved in Europe in 2002, registered with a class III CE mark as a medical device. It received FDA approval in 2014 for apheresis platelets. INTERCEPT uses a combination of UVA illumination and the photosensitive psoralen compound amotosalen to achieve broad-spectrum pathogen inactivation [45]. Amotosalen is added to the apheresis platelet component and exposed to 3 J/cm^2^ UVA light for 3–5 min, and residual amotosalen and photoproducts are removed using a compound adsorption device. The process is typically performed at a blood collection facility within 24 h of platelet donation.

Psoralens are found naturally in some foods and have an affinity for nucleic acids. Amotosalen is a synthetic psoralen that intercalates into DNA and RNA. It forms strong covalent bonds only after UVA light, resulting in formation of monoadducts and cross-linking of DNA and RNA [45] and irreversibly blocking replication and repair. This reaction occurs in an oxygen-independent manner. Amotosalen was selected for its activity against a wide range of bacteria (Gram-positive and Gram-negative) and viruses (enveloped and non-enveloped) [46, 47] without compromising platelet function. It passes through cellular membranes, bacterial walls, and viral envelopes but crucially does not interact with other cellular components [45]; therefore, the functional characteristics of blood components are maintained. Amotosalen also has activity against residual leukocytes, helping to reduce risk of transfusion-associated graft-versus-host disease and infection with intracellular pathogens such as cytomegalovirus and Epstein–Barr virus for which leukocytes are a natural reservoir. Treatment of platelet concentrates with INTERCEPT technology therefore reduces, and may replace, the need for cytomegalovirus serology, bacterial detection, and gamma irradiation. The clinical indications for INTERCEPT-treated platelets remain the same as for conventional platelet concentrates.

The safety and efficacy of platelets treated with INTERCEPT before transfusion have been demonstrated in multiple studies [48–50]. A 7-year, multinational, prospective, HV study (19,175 INTERCEPT-treated platelet transfusions in 4067 patients) found a low rate of acute transfusion reactions and a safety profile consistent with that previously reported for conventional platelet components [51]. Combined national HV data from France and Switzerland showed that 310,362 INTERCEPT-treated platelet transfusions were associated with no septic reactions or deaths, whereas about 2.5 million conventional (untreated) platelet transfusions led to 62 STRs and 11 deaths [4]. In a US phase III clinical trial, INTERCEPT-treated platelets met the primary endpoint of non-inferiority for the incidence of grade 2 bleeding when compared with conventional platelets [52]. A retrospective comparative effectiveness study in about 1700 patients found that pathogen inactivation with amotosalen and UVA illumination did not significantly affect blood product utilization or the efficacy and safety profile of platelet transfusions in adult and pediatric patients (mostly cardiac surgery and H/O patients) [53]. These findings show that pathogen inactivation of platelet concentrates using amotosalen and UVA illumination is compatible with the routine operations of a large tertiary care hospital.

### Mirasol^®^ Pathogen Reduction Technology System

The Mirasol^®^ Pathogen Reduction Technology system (Terumo BCT, Lakewood, CO, USA) is not FDA-approved (starting phase III trials) but is approved in selected markets, including some European countries. Mirasol is also based on supplementation with a photosensitive agent in combination with UV illumination, primarily UVB, and relies heavily on the generation of reactive oxygen species as the mechanism of action. This process reduces pathogen load and inactivates residual leukocytes. Rather than a synthetic psoralen, the Mirasol system uses riboflavin, a naturally occurring vitamin (B_2_), as a photosensitizer that promotes oxidation of nucleic acids. In conjunction with broad-spectrum UV light (60% of UVA, 100% of UVB, and 20% of UVC), this causes nucleic acid chain modifications that are selectively targeted to guanine bases [54]. The resulting damage to DNA/RNA is irreversible because of the inhibition of replication and repair processes [54]. Administration of riboflavin by multiple routes has been shown to be safe and no new chemicals are introduced into the blood since the photoproducts generated from its breakdown are physiological metabolites that are present in human blood [54].

The Mirasol system is effective against a range of bacterial, viral, and parasitic infections [54, 55]. Treatment with Mirasol demonstrated an overall effectiveness of 98% against 20 clinically relevant bacterial strains when present at very low bacterial concentrations characteristic of contamination at the time of collection; effectiveness was 91% at concentrations more typical for slow-growing bacteria at the timing of Mirasol treatment [55]. However, biofilm-producing strains such as *Staphylococcus epidermidis* may not be completely inactivated following riboflavin/UV treatment of platelet concentrates [56]. Some studies indicate preservation of platelet function following Mirasol treatment [57] and no difference in the subsequent utilization of platelet and red blood cell products relative to reference platelets [58]. Others have reported significant effects of riboflavin/UV treatment on platelet function, production of reactive oxygen species, and oxidative damage [59, 60]. In a randomized controlled trial of 118 patients with thrombocytopenia secondary to chemotherapy, Mirasol-treated platelets exhibited a safety profile comparable to reference platelets but failed to meet noninferiority criteria based on corrected count increment [58].

### THERAFLEX^®^ UV-Platelets

The THERAFLEX^®^ UV-Platelets system (MacoPharma, Mouvaux, France) is still undergoing clinical development and is not currently licensed in any market. THERAFLEX does not rely on supplementation of blood products with a photoreactive agent. This system achieves pathogen reduction/inactivation using short-wave UVC illumination alone. Targeted UVC irradiation (254 nm wavelength) induces the formation of pyrimidine-based dimers and global lesions within nucleic acid strands [61]. Illumination is performed on both sides of the platelet bag and typically requires less than one minute to achieve a biologically active dose of UVC; this is coupled with intense agitation to ensure uniform treatment [61]. The THERAFLEX system is effective at inactivating a variety of bacterial and viral species [61, 62], including emerging viruses [63]. Limited effects of UVC treatment on platelet quality have been reported [64], although the possible impact of the hard agitation remains to be determined. A phase I study in healthy individuals has shown that treated platelets are well tolerated [65].

## Potential limitations of mitigation strategies

Each strategy described above comes with unique limitations, many of which are described above and summarized here. Testing-based strategies are susceptible to sampling errors, bacterial growth phase lag, aerobic versus anaerobic targeting, and biofilm detection, and they place restrictions on product availability and shelf life. This can lead to fatal outcomes. Recently, two case reports described three deaths due to bacterially contaminated platelets. The authors stated that although all current procedures were followed, the risk of transfusion-transmitted infections and fatality remains [20]. Pathogen reduction/inactivation systems use different mechanisms and thus show different bacterial inactivation results; no pathogen reduction/inactivation system is effective against all pathogens. Furthermore, pathogen reduction is not currently available for all platelet collections (for example, whole blood-derived, 7-day), although development is ongoing.

## Current guidance and future perspectives

Despite advances in strategies to address the risks associated with platelet transfusions, the potential for bacterial contamination remains. Recently, the FDA published draft guidance for blood centers and transfusion services on how to mitigate the risk of bacterial contamination of platelets [4]. These recommendations focus on pathogen reduction/inactivation technologies or bacterial testing of platelets for transfusion. Both approaches reduced the incidence of septic reactions associated with platelet transfusions in a large, retrospective, international HV study [41]. The AABB proposed that the FDA draft guidance go further and mandate the routine implementation of enhanced, proactive, safety strategies using bacterial testing or pathogen reduction with FDA-approved technologies [66], a view shared by others [67].

Although current pathogen reduction/inactivation technologies are effective against most bacterial contaminants of platelets, they are not particularly effective against bacterial spores, and unknown and emerging pathogens are an ongoing challenge with some technologies. Progress in pathogen reduction/inactivation technologies continues, including the clinical development of technologies for treating whole blood, red blood cells, and cryoprecipitate [68, 69]. Research efforts to further develop and validate technologies for pathogen reduction/inactivation and bacterial detection will be aided by the recent enlargement of the World Health Organization international repository for platelet transfusion-relevant bacterial reference strains [70]. Minimizing risks associated with bacterially contaminated platelets will require improvements in surveillance and avoidance strategies to prevent contamination and, in detection methods for identifying contaminated platelet units, toward the ideal of readily available pathogen-safe platelets.

## Conclusions

Bacterial contamination remains a substantial risk to patients requiring platelet transfusions. In addition to H/O patients, critically ill and surgical patients may be at particular risk. Significant progress has been made to minimize this risk, and further research and technological developments are ongoing. Historical avoidance and screening strategies have reduced but not eliminated the threat of transfusion-related bacterial infections. Recent and future regulatory approval of technologies for pathogen reduction and inactivation provides further methods to reduce infections acquired through transfusion of platelets.

## Abbreviations

AABB: American Association of Blood Banks
CDC: Centers for Disease Control and Prevention
FDA: US Food and Drug Administration
H/O: Hematology/oncology
HV: Hemovigilance
ICU: Intensive care unit
PGD: Platelet Pan Genera Detection
STR: Septic transfusion reaction
TTBI: Transfusion-transmitted bacterial infection
UV: Ultraviolet
